# USAT: a bioinformatic toolkit to facilitate interpretation and comparative visualization of tandem repeat sequences

**DOI:** 10.1186/s12859-022-05021-1

**Published:** 2022-11-19

**Authors:** Xuewen Wang, Bruce Budowle, Jianye Ge

**Affiliations:** 1 Center for Human Identification, Health Science Center, University of North Texas, Fort Worth, TX USA; 2 Department of Microbiology, Immunology, and Genetics, University of North Texas Health Science Center, Fort Worth, TX USA

**Keywords:** Tandem repeat, Allele comparison, DNA, Haplotype, Forensics STR, Genome comparison

## Abstract

**Background:**

Tandem repeats (TR), highly variable genomic variants, are widely used in individual identification, disease diagnostics, and evolutionary studies. The recent advances in sequencing technologies and bioinformatic tools facilitate calling TR haplotypes genome widely. Both length-based and sequence-based TR alleles are used in different applications. However, sequence-based TR alleles could provide the highest precision in characterizing TR haplotypes. The need to identify the differences at the single nucleotide level between or among TR haplotypes with an easy-use bioinformatic tool is essential.

**Results:**

In this study, we developed a Universal STR Allele Toolkit (USAT) for TR haplotype analysis, which takes TR haplotype output from existing tools to perform allele size conversion, sequence comparison of haplotypes, figure plotting, comparison for allele distribution, and interactive visualization. An exemplary application of USAT for analysis of the CODIS core STR loci for DNA forensics with benchmarking human individuals demonstrated the capabilities of USAT. USAT has user-friendly graphic interfaces and runs fast in major computing operating systems with parallel computing enabled.

**Conclusion:**

USAT is a user-friendly bioinformatics software for interpretation, visualization, and comparisons of TRs.

**Supplementary Information:**

The online version contains supplementary material available at 10.1186/s12859-022-05021-1.

## Introduction

Genomic sequence variation between and among individuals within and between species is of genetic and practical significance. Tandem repeats (TRs), a type of genomic variation, comprise a few to hundreds of tandemly repeated sequences in the genome [1, 2]. A TR can vary in the number of repeats between species and among individuals of the same species [3]. TRs are classified into short TRs (STRs), also known as microsatellites, and variable number tandem repeats or minisatellites. In particular, STRs usually contain a repeat motif, ≤ 6 base pairs (bp) in length, are widely dispersed in genomes and compose up to ~ 1–3% of most eukaryotic genomes [4–6]. TRs were known before the genomic era and were used as genetic markers (e.g., STR markers). However, it is still challenging to complete a genome-wide analysis of TRs and understand TR significance in living organisms due to the complexity of TRs. More recently, our understanding of TRs has been increasingly enriched with the advances in better-assembled genomes, high throughput DNA sequencing technologies, and bioinformatics analyses [2, 7–10]. With the complex variabilities and high discrimination powers, TR markers have been widely used in population genetic analyses, forensic identification, molecular breeding, and selection [5, 6, 11–14]. In addition, TR variations are known to associate with neural diseases, such as Alzheimer’s, obesity, and cancers via regulating proximal gene expression [7, 15]. Also, STRs are the core markers of forensic DNA applications and are used in almost all forensic DNA databases, such as the FBI’s Combined DNA Index System (CODIS) database [16].

In many studies and practices, the lengths of TR alleles are used, while the detailed sequence of alleles is ignored. For example, a forensic STR allele is typically recorded as the number of repeats or length-based sizes (e.g., 10.1 for an allele comprising ten repeats plus one additional base). This operationally-defined designation is due to the limitations of traditional technologies, with which the variants of TR are detected by Sanger sequencing or by measuring the lengths of DNA fragments during separation by capillary electrophoresis (CE). TR alleles with the same length are treated as the same alleles, although they may have different sequences. The higher resolution of TR alleles can be important for a wide range of applications and currently has not been fully captured.

TR alleles can be reported as sequence variants or haplotypes using next-generation sequencing (NGS) technologies with higher confidence and lower cost per base pair than traditional methods [17]. Bioinformatic tools have been developed to detect TR haplotypes from sequence datasets, such as STRait Razor [18, 19], HipSTR [2] and FDSTools [20]. These software programs can detect both length-based and sequence-based alleles. Each STR haplotype (i.e., sequence) contains rich information such as the number of repeats of a basic motif, and additional point mutations such as single nucleotide polymorphism (SNP) and insertions/deletions, if present. However, it is difficult to directly or visually identify the differences between TR haplotypes due to their repetitive nature, especially for complex haplotypes. In addition, some repeat expansions of disease-associated TRs may be very long and contain multiple types of variants, which could further complicate comparisons. Multiple sequence alignment tools like MAFFT can compare highly similar sequences and identify the difference between sequences [21]. However, these tools are usually developed for a general comparing purpose.

The latest submission requirements of CODIS [16] have begun to accept the STR haplotype sequences. A conversion between sequence-based alleles and length-based alleles (i.e., the latter being the current allele designations in the CODIS system) is needed for backward compatibility purposes. Also, in many forensic mixture cases, a mixture profile typically contains multiple STR allele haplotypes from multiple contributors, and an effective comparison between these haplotypes could facilitate deconvolution of the profile.

In this study, we developed an end-user-friendly graphic bioinformatic software, Universal STR Allele Toolkit (USAT), which provides a comprehensive set of functions to analyze and visualize TR alleles, including the conversion between length-based alleles and sequence-based alleles, nucleotide comparison of TR haplotypes, an atlas of allele distributions, interactive data filtering, data formatting, and visualization in parallel computing with a graphic user interface (i.e., no command line is needed). The latest forensic recommendations for DNA forensics [22, 23] were followed. In general, USAT facilitates the deep analysis of TR haplotypes and TR allele interpretation. The software can run in the major operating systems, including Windows, macOS, and Linux.

## Methods

### Data format of TR alleles and TR locus

**Fig. 1:**
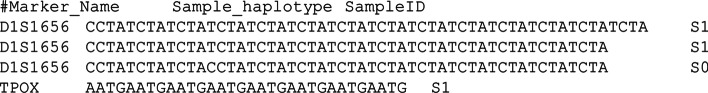
The format of the input file with TR sequence data. The data consists of multiple lines of haplotypic information in a plain text format. Each line is tab-delimited three fields of marker name, DNA sequence of the haplotype, and sample IDs. The # line is for comment

Two input datasets in a plain text format are required, including a TR sequence file and a configure file for a tandem repeat locus or loci in the BED format (https://useast.ensembl.org/info/website/upload/bed.html). The sequence file contains a tab-separated locus or marker name, the DNA nucleotide sequence of a TR haplotype at this allele, and the name of a DNA sample (Fig. 1). One sample usually has multiple alleles (i.e., sequences) at each locus. This sequence file can contain the information of multiple alleles, and each line is for one allele. Lines starting with # will be used as comment information only and will be ignored during processing. The configure file in BED format contains the information of the required name of a marker, the length in bps of the basic motif period, and the excluded length of nucleotides (termed as inner offset) in bps for length-based forensic allele size designation while other fields are can be filled with value 1 or zero if unknown. An exemplary BED configure file for the CODIS 20 core STRs with coordinates of human genome assembly GRCh38 is distributed with the software (Additional file 1: Dataset 1).

### Algorithm for TR allele size converting

TR size is well defined and used in the forensic practice and databases. For applications in forensics, the sequence-based TR alleles can be converted to length-based TR alleles, representing the number of repeats, for backward compatibility with the forensic DNA databases. The calculation approach is based on the latest recommendation [22, 23], and the alleles were formulated with the equations below. The TR allele size usually contains both an integer part and a fractional part, separated by a dot (e.g., 5.1). The fraction part is typically omitted if it is zero.

$$\begin{gathered} Integer part of allele size = Floor\left( {\frac{Allele length - Internal offset}{{Period}}} \right) \hfill \\ Fractional part of allele size = Remainder\left( {Allele length - Internal offset, Period} \right) \hfill \\ \end{gathered}$$

in which the Floor (x) is the function to calculate the greatest integer less than or equal to x; Remainder (x, y) is the remainder of x divided by y; the allele length is the total number of nucleotide bases of an allele; the internal offset is the number of bases that need to be excluded in counting the length-based allele, and the period is the length in base pair of a repeat motif. For example, for a TR with a motif of ATCG (period = 4) and an internal offset of 2, the integer part of the allele size of a sequence allele “ATCGATCGggATCGA” (“gg” as internal offset sequences) would be Floor((15 − 2)/4) = 3, and the factional part is the remainder of (15 − 2)/4, which is 1, and thus the length-based allele size would be 3.1. To view and compare the haplotype sequences, the length in base pair and the number of repeats are calculated with this formula with the locus configuration information in the BED input file.

### Interactive view and sequence alignment

USAT was programmed with Java JDK 16 (https://www.oracle.com/). JFreeChart Java library (version 1.5.3) (www.jfree.org) was used to plot figures. To compare the haplotype sequences, the user-selected haplotype sequences were dynamically retrieved from an interactively viewing table to construct the input file for MAFFT [21]. MAFFT is a multiple sequence aligner which is fast and good for comparing up to hundreds of DNA sequences with high similarity. To compare the whole TR sequences, the global alignment using Needleman-Wunsch algorithm in MAFFT is used in USAT [21]. The alignment of multiple sequences in the Clustal format [24] is then accepted by USAT and displayed in an interactive and editable window for customized fine-tuning, which enables to adjust the possible multiple alignment results of tandem repeat sequence.

### Workflow of USAT

**Fig. 2:**
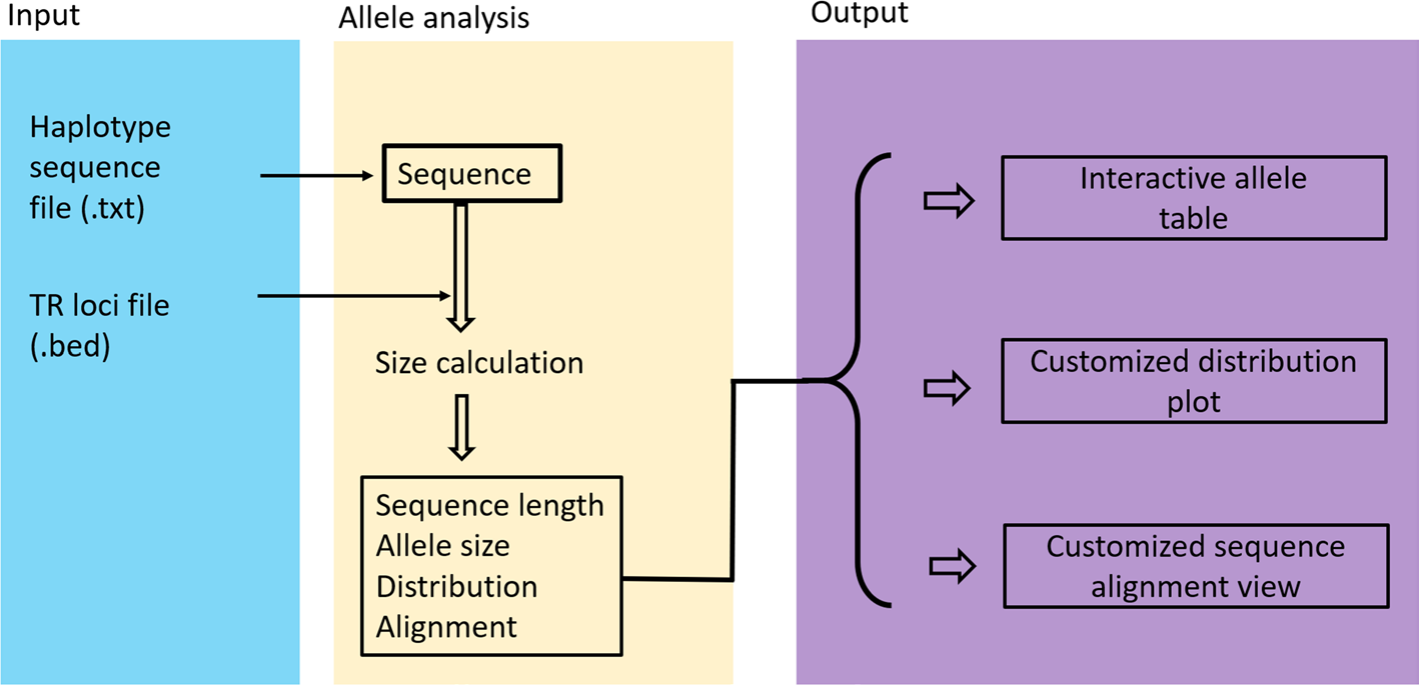
The workflow of USAT software. Three major modules of the USAT workflow are input, allele analysis, and output. The input module takes a DNA haplotype sequence file in tabular plain text format and a BED file describing the details of the tandem repeat (TR) loci. The haplotype is used to count the length in base pairs and the number of repeats (allele size) based on locus position information in the BED file. All haplotypes and calculated data are then used in displaying in an interactive table and plotting a graphic distribution requested by the user. Haplotypes of interest are aligned to identify the detailed difference between/among the haplotypes

USAT takes the TR sequences in a plain text file and TR loci configure information in a BED formatted plain text file as input to calculate the length of each haplotype sequence in nucleotide base pairs (bps) and the number of repeats (allele sizes) using the equation described in the method section (Fig. 2). The input of TR sequences can be easily reformatted from the output of existing tools, such as the text output from STRait Razor [18, 19] and FDSTools [20], or VCF output from HipSTR [2]. All TR data are then displayed by USAT in an interactive table for viewing, sorting, filtering, reformatting via dragging, and saving to a result file. Interactive graphic plot(s) can be generated to show an atlas of size or length distributions for selected alleles. Multiple selected sequences can be aligned with integrated MAFFT [21] and visualized for TR sequence comparisons, with identity marked. USAT was programmed with Java and tested in the major operating systems, such as Windows 10 (version 21H1), macOS (version 11.6), and Ubuntu Linux (version 20.4). All functions are integrated into a user-friendly graph interface, and only mouse clicks are needed to run all analyses (Fig. 2). Overall, USAT is a user-friendly software for any end-user with minimum bioinformatic skills. A command-line interface of USAT calculator for converting the sequence-based alleles to length-based alleles also is provided for software developers or other pipelines as needed.

### Testing data and speed test

The haplotype sequences of 20 CODIS core STR loci in publicly available benchmark human samples HG002 (son) and HG003 (father) were retrieved from the Genome In A Bottle (GIAB) project. The same sequences were also generated with the ForenSeq kit in a previous study [25]. All these sequences were manually verified in a sequence alignment. The haplotype sequences of each STR locus were prepared in tabular plain text format according to the required format as an input for USAT (Additional file 1: Dataset 2 and 3). The running speed of USAT was tested for analyzing all CODIS core STR alleles with two computing threads on a Windows 10 system with an i7-10875H processor and 32 G memory and Linux Ubuntu 20.4 system with an Xeon Gold 6226R and 256 G memory.

## Results

### Overview of the graphic software USAT

**Fig. 3:**
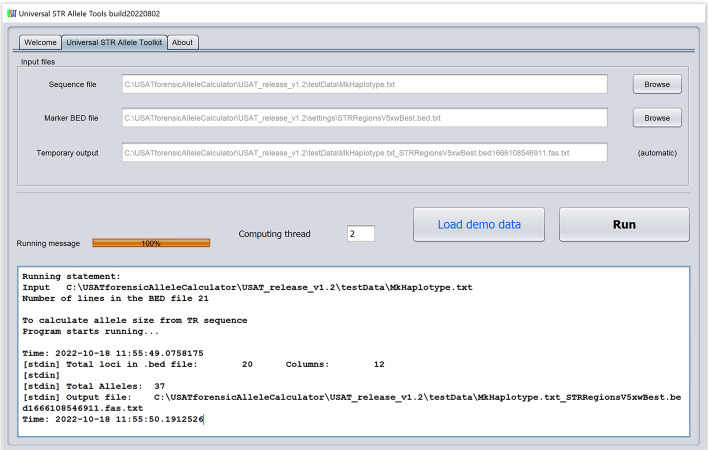
The input interface of USAT

USAT graphic interface takes two input files in plain text format, which are the TR sequence file and a configure file in the BED format for a tandem repeat locus or loci. The input files are easy to be selected only by mouse clicks (Fig. 3).

**Fig. 4:**
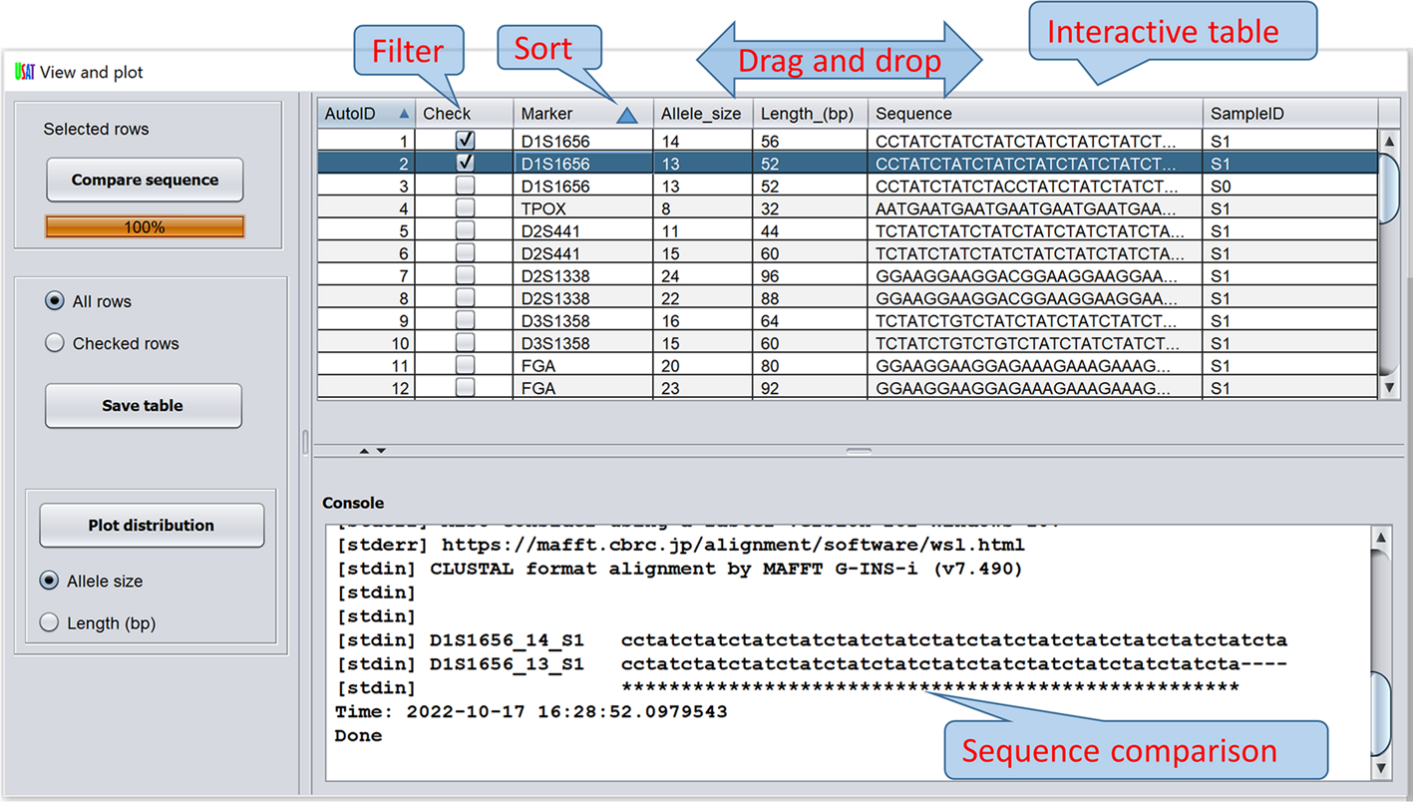
Interactive table for tandem repeat features and haplotype comparison. The top panel is an interactive table for viewing, filtering, and sorting. The bottom panel shows the sequence comparison between selected haplotypes of interest. The callouts in red letters are added as annotations for better understanding, not from the software

USAT reports the haplotype length, the number of repeats or allele size, haplotype for each locus and the sample name in an interactive table (Fig. 4). In the table, data in each column could be sorted in ascending or descending order via just mouse clicks on the column header. The order of each column could be changed by dragging and dropping the column header. This capability could change the current format into a desired format in the output file, which can facilitate further preferred formatting for subsequent analysis (e.g., preparing for submissions to CODIS or STRidER at https://strider.online/). Figure 4 demonstrates the application of USAT for reporting alleles of the 20 CODIS core STR loci of benchmark human sample HG002 in the GIAB project (https://www.nist.gov/programs-projects/genome-bottle).

To compare the haplotypic differences between multiple alleles, USAT aligns DNA sequences by integrating the tool MAFFT [21] which is capable of aligning hundreds of sequences, and displays the alignment by showing the difference and consistency with markers of asterisk (complete identity), colon (strong similarity), period (weak similarity), and dash for insertion/deletion, following the Clustral format (www.clustal.org). Figure 4 shows the identity in alignment and the differences of allelic sequences of marker D1S1656 of a human reference.

**Fig. 5:**
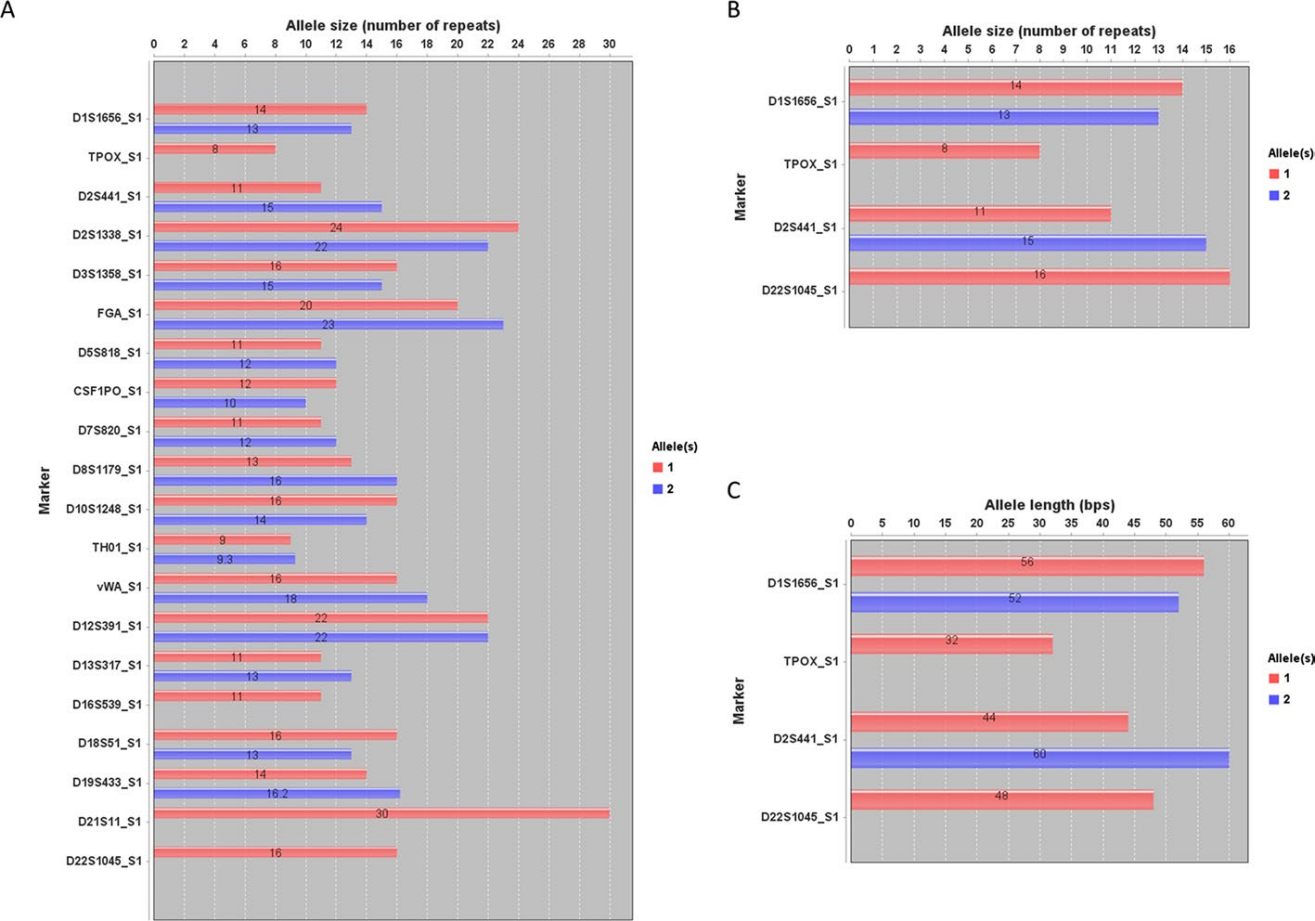
Atlas and distribution plots of tandem repeat alleles. Plots generated by USAT for TR alleles of CODIS core STR markers in the human benchmark sample HG002 from the Genome In A Bottle project. The number on the bar is the detailed size value of the x-axis of an allele. **A** A bar plot showing the entire atlas of alleles of all 20 CODIS core STR markers. **B** A plot showing the comparison of allele sizes of selected markers. **C** A plot showing the comparison of allele length in base pairs of selected markers. The x-axis shows the number of tandem repeat units or allele length in base pairs. The label next to the y-axis shows the name of CODIS STR markers. The name of each bar group is encoded as marker/locus name and sample name joined by an underscore

To view the atlas of allele distributions of targeted markers, interactive bar figure(s) can be plotted in USAT for any selected markers. This display enables an overviewing of the atlas of selected alleles and comparison by allele sizes or lengths. The end-user could zoom in or out of the figure and save the plot for any purpose (e.g., publication or report). For example, Fig. 5 shows the atlas of a complete set of 20 CODIS core loci in human sample HG002 and also the comparison of the allele size or length in bps of alleles of the selected markers. Overall, USAT provides the functions to visualize and interpret both length-based and size-based TR alleles.

### Speed and functional comparison with other tools

USAT is ultrafast with parallel computing enabled. It took less than one second to analyze the length and size calculation and display all TRs at 20 CODIS core STR loci of the benchmark human sample HG002. The multiple haplotype sequences comparison step takes ~ 200 mili-seconds and around 9 s in Ubuntu Linux 20.4 and Windows 10 system, respectively. STRait Razor and FDSTools are widely used for forensic STR allele analysis. Compared with these tools in terms of TR haplotype analysis, USAT is universal and flexible for powerful sequence comparison, graphic comparison and visualization (Table 1).

**Table 1:** Comparison of USAT with other tools

	USAT	STRait razor online	FDSTools
Input TR data	Universal	Self only#	Self only#
Universal TR size conversion	Yes	Forensic*	Forensic*
Plot of allele sizes, length	Yes	Size only	Size only
Comparison of distribution atlas	Yes	No	No
Haplotype sequence comparison	Yes	No	No
Interactive figure	Yes	No	No
Haplotype reformatting	Yes	No	No
Parallel computing	Yes	No	No
Computing system	Windows, Linux, macOS	Windows, Linux	Windows, Linux, macOS
User interface	Graphic	Web page R shiny	Command
Easy to use	Very easy	Easy	Training needed

^#^ the conversion is for software designed forensic kit only; * only for forensic data generated from the software itself

### Application of USAT for TR comparison

**Fig. 6:**
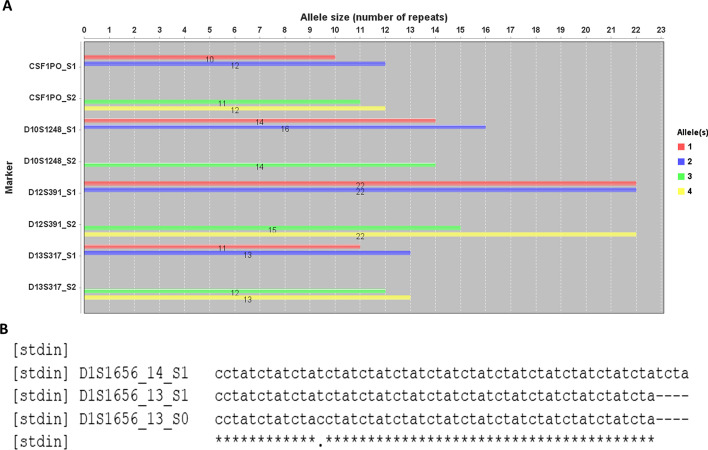
Application example of USAT for TRs between samples. **A** An example showing the difference of TR allele sizes between human reference individual HG002 (S1, son) and HG003 (S2, father) from the Genome In A Bottle project. The name of each bar group is encoded as a marker/locus name and sample name joined by an underscore. **B** An alignment showing the difference between TR haplotypes, where the dot position suggested allele D1S1656_10_S0 has a simulated mutation in the TR sequence. The name of each sequence is encoded with a marker/locus name, allele size, and sample name joined by an underscore

The above sections demonstrated TR comparisons within a sample or an individual with USAT. To demonstrate the application of USAT for TR comparisons between individuals, TRs from human reference HG002 (son, S1) and HG003 (father, S2) in GIAB were formed as a mixture, and then fed into USAT for analysis. Results of comparing four CODIS STR loci demonstrating a clear TR difference and one copy of TR inheritance between a son and a father are shown in Fig. 6A. To test and visualize the capability of USAT for analyzing mutations, a mutation within the TR allele of CODIS marker allele D1S1656-13 was simulated from sample S1 and marked as S0. The TR sequence comparison successfully showed an expected mutation site in the alignment marked as a dot (Fig. 6B), which helps easy visualization of the difference between alleles.

## Discussion

Genomic sequence variations contain genetic and evolutionary clues of both theoretical and practical values. Increasing amounts of sequence data and studies have enhanced the discovery of sequence-based TR haplotypes [2, 8, 12, 19]. Further analysis within TR haplotypes could provide additional understanding and interpretation of TR variation and TR’s role in organisms. For example, 25 new sequence variants from 15 CODIS loci were found in an Austrian massive parallel sequencing dataset of 247 reference human samples via sequencing targeted STR loci compared with via a length-based CE method [26]. Such variants were undetectable with traditional CE methods, which do not accurately reflect the underlying sequence genotypes. Existing TR associated tools mainly focus on mining or phase TR sequences out of other sequences and report only the length and TR haplotype (e.g., HipSTR [2]), or the number of repeats and TR haplotype (e.g., STRait Razor [19]). Here, our novel USAT software fills the gap for comprehensive characterization, visualization and comparison of TR sequences.

We choose the tab-delimited plain text format for the input sequence file for USAT because this format is much close to TR outputs from other existing upstream tools, e.g., a text output from STRait Razor [19], FDSTools [20] and VCF file from HipSTR [2] etc. This enables minimum data reformatting. We added an extra field called “Sample ID” in the last field to label the DNA sources, which is helpful for comparison for the same locus between samples. The BED input file with marker locus information is compatible for the widely used BED format in other locus viewers like Genome Browser (https://genome.ucsc.edu/). The specified BED format only needs three additional mandatory values of the basic motif length, inner offset and sample ID while others can be just set as other values, e.g., one or zero. In addition, this required BED format for USAT is fully compatible with STR locus information in forensics community and databases, like CODIS.

Visualizing and comparison of TR haplotypes have been of research interest for decades. A wet–lab experimental method using a fluorescence reporter to detect and visualize TR mutation in bacterium was reported [27]; however, it is limited to a specific STR locus and is low throughput. Lots of TR detection software tools have been developed, such as TRfinder [28], HipSTR [2], GMATA [6], TRtools [29], Dot2dot [30], STRait Razor [18, 19], and others reviewed by Lim et al. [31]. Among those, HipSTR is the most advanced tool currently. However, the existing tools can’t compare the TR haplotypes, which is a challenge. Some tools may identify and phase two haplotypes, e.g., HipSTR. The latest sequencing technologies and bioinformatics tools make the TR haplotype available. TR haplotypes are highly similar in sequence but are different from each other. The REViewer provides haplotype-resolved visualization of sequencing read alignments around TR regions but no comparison between haplotypes [32]. Three or more TR haplotypes may commonly present in a DNA mixture from multiple individuals or cancer cells for a given TR locus. Here, USAT can take output haplotypes from existing tools, and then compare two or more TR haplotypes via multiple sequence alignment. Thus, USAT provides an extended solution to characterize TR haplotypes deeply.

USAT provides several features for TR applications. The informative TR results generated by USAT are able to facilitate individual identification, TR comparison, marker selection, and further TR marker development (e.g., selecting appropriate TR loci for specific purposes). USAT also provides a direct viewing of the length distribution of TR sequences, which may be used, for example, in TR-associated diagnostic screening of specific diseases. For example, the variation of long tandem repeat loci in gene *ATXN10* and *C9orf71* is associated with Parkinson’s disease and amyotrophic lateral sclerosis, respectively [14, 33]. In addition, USAT can provide the detailed descriptions of sequence-based alleles for the STR Sequencing Project (STRSeq) (https://www.ncbi.nlm.nih.gov/bioproject/380127). TRs are widely used for DNA barcoding in many evolution diversity studies, and thus USAT may also be used in biodiversity investigation and discovery of novel species [34]. Some conflictions are common based on the phylogenetic results from the length only based TR comparison in previous research, which may be resolved by USAT once the TR sequences are available and analysed by USAT. The completion of T2t_CHM13 human genome assembly makes rich TR information available at the segmental duplication (SD) regions [35]. Long sequencing reads make the identification of new TR haplotypes for SD regions. Telomere, consisting of mostly TRs, becomes shorter with increasing life span and associated with disease [36] [35]. The recently available complete telomere genome assembly unveils previous unknown knowledge, e.g. evolutionary genomics and genetics clue in sex chromosomes [37, 38]. Once the TRs in telomere are available, USAT provides a solution to view and compare the differences between telomeres so help to decode the genetic clues for research and diagnosis. Any TR haplotypes can be input into USAT for deep comparison. Thus, USAT allows us to improve the TR comparison and characterization for TR haplotypes in human genome or other species.

More computing threads in general can speed up the analysis in USAT. However, if a dataset is small, two threads in the default setting should be sufficient to obtain results in seconds. In addition, while the alignment of haplotypes may not be in the best format due to multiple possibly acceptable alignments, an additional editing function in the alignment output is enabled to allow users to adjust the user preferred alignment as needed. Here, we want to point out that USAT takes the TR haplotype sequences as the input, instead of raw TR sequencing reads from high throughput sequencers. Millions of TR raw sequencing reads may not good for aligner MAFFT. In this case, mapping the TR raw sequencing reads to reference genome and assemble the reads to haplotypes is required before feeding into USAT. In addition, if a user wants to generate a left-align format alignment, it is better to add several non-TR bases to the left of each TR haplotype in the put sequence file so USAT is highly to generate a left-aligned alignment.

TRs are widely used by a range of researchers with various backgrounds and varying bioinformatic skills. Thus, ease of use for end-users is a very important feature of any bioinformatic tool. USAT was specifically designed for users with limited knowledge and skills in bioinformatics. It provides user-friendly graphic interfaces that can be easily adopted by end-users with minimum effort.

## Conclusions

USAT is a user-friendly graphic bioinformatics software for sequence comparison, allele size conversion, plotting, and visualization of genomic tandem repeat haplotypes. USAT will serve as a universal tool for precision analysis of TR haplotypes generated from other tools in forensics, disease diagnosis, evolutionary genomics, and other breeding areas.

## Supplementary Information

**Additional file 1. Dataset 1. **An exemplary BED file for 20 core STRs of human in CODIS.** Dataset 2. **Input data of haplotype for HG002 (S1).** Dataset 3. **Input data of haplotype for HG003 (S2).

## Data Availability

The software and data used in the current study is freely available on https://github.com/XuewenWangUGA/USAT or https://github.com/Ge-lab and supplementary information. The data used in this study is included in the a subfolder called testData.

## Abbreviations

CE: Capillary electrophoresis
CODIS: Combined DNA index system
DNA: Deoxyribonucleic acid
NGS: Next generation sequencing
SNP: Single nucleotide polymorphism
STR: Short tandem repeats
TR: Tandem repeat
USAT: Universal STR allele toolkit
VCF: Variant call format
