# Supplementary material for: USAT: a bioinformatic toolkit to facilitate interpretation and comparative visualization of tandem repeat sequences
Source: BMC Bioinformatics. 2022 Nov 19;23:497. doi: 10.1186/s12859-022-05021-1 (PMC9675219; doi:10.1186/s12859-022-05021-1)
Supplement: Supplementary file 1 — Additional file 1. Dataset 1. An exemplary BED file for 20 core STRs of human in CODIS. Dataset 2. Input data of haplotype for HG002 (S1). Dataset 3. Input data of haplotype for HG003 (S2). [file 12859_2022_5021_MOESM1_ESM.docx]

## **USAT: a Bioinformatic Toolkit to** **Facilitate Interpretation and Comparative Visualization of Tandem Repeat Sequences**

##

Supplementary data:

# An exemplary BED file for 20 core STRs of human in CODIS

Chrom ChromStart ChromEnd Name Left_offset Right_offset Basic_motif_period Ref_hap_length Motif Ref_allele Inner_offset Min_stutter_threshold

chr1 230769615 230769683 D1S1656 3 3 4 68 CCTA [TCTA]n TCA [TCTA]n 17 0 0.1

chr2 218014858 218014950 D2S1338 3 3 4 92 [GGAA]n GGAC [GGAA]n [GGCA]n 23 0 0.1

chr2 1489652 1489684 TPOX 3 3 4 32 [AATG]n 8 0 0.1

chr2 68011946 68011994 D2S441 3 3 4 48 [TCTA]n TCA [TCTA]n 12 0 0.1

chr3 45540738 45540802 D3S1358 3 3 4 64 [TCTA]n [TCTG]n [TCTA]n 16 0 0.1

chr4 154587735 154587823 FGA 3 3 4 88 [GGAA]n GGAG [AAAG]n AGAA AAAA [GAAA]n 22 0 0.1

chr5 123775555 123775599 D5S818 5 3 4 44 [ATCT]n 11 0 0.1

chr5 150076323 150076375 CSF1PO 3 3 4 52 [ATCT]n 13 0 0.1

chr7 84160225 84160277 D7S820 3 3 4 52 [TATC]n 13 0 0.1

chr8 124894864 124894916 D8S1179 3 3 4 52 [TCTA]n [TCTG]n [TCTA]n 13 0 0.1

chr10 129294243 129294295 D10S1248 3 3 4 52 [GGAA]n 13 0 0.1

chr11 2171087 2171115 TH01 3 3 4 28 [AATG]n ATG [AATG]n 7 0 0.1

chr12 5983976 5984044 vWA 3 4 4 68 [TAGA]n [CAGA]n TAGA 17 0 0.12

chr12 12297019 12297095 D12S391 6 4 4 76 [AGAT]n GA T [AGAT]n [AGAC]n AGAT 19 0 0.1

chr13 82148024 82148068 D13S317 3 3 4 44 [TATC]n 11 0 0.1

chr16 86352701 86352745 D16S539 3 3 4 44 [GATA]n 11 0 0.1

chr18 63281666 63281738 D18S51 3 3 4 72 [AGAA]n AG 18 0 0.1

chr19 29926234 29926298 D19S433 3 3 4 64 [CCTT]n ccta [CCTT]n cttt [CCTT]n 8 8 0.1

chr21 19181972 19182099 D21S11 3 3 4 127 [TCTA]n [TCTG]n [TCTA]n ta [TCTA]n tca [TCTA]n tccata [TCTA]n TA [TCTA]n 29 11 0.1

chr22 37140286 37140337 D22S1045 3 3 3 51 [ATT]n ACT [ATT]n 17 0 0.1

# Input data of haplotype for HG002 (S1)

#note: the third sequence for S0 is an simulated sequence with a mutation relative to the 2^nd^ sequence.

#CODIS core STR loci for HG002

#Marker_Name Sample_haplotype SampleID

D1S1656 CCTATCTATCTATCTATCTATCTATCTATCTATCTATCTATCTATCTATCTATCTA S1

D1S1656 CCTATCTATCTATCTATCTATCTATCTATCTATCTATCTATCTATCTATCTA S1

D1S1656 CCTATCTATCTACCTATCTATCTATCTATCTATCTATCTATCTATCTATCTA S0

TPOX AATGAATGAATGAATGAATGAATGAATGAATG S1

D2S441 TCTATCTATCTATCTATCTATCTATCTATCTATCTATCTATCTA S1

D2S441 TCTATCTATCTATCTATCTATCTATCTATCTATCTATCTATCTATCTATTTATCTATCTA S1

D2S1338 GGAAGGAAGGACGGAAGGAAGGAAGGAAGGAAGGAAGGAAGGAAGGAAGGAAGGAAGGAAGGAAGGAAGGAAGGCAGGCAGGCAGGCAGGCAGGCA S1

D2S1338 GGAAGGAAGGACGGAAGGAAGGAAGGAAGGAAGGAAGGAAGGAAGGAAGGAAGGAAGGAAGGCAGGCAGGCAGGCAGGCAGGCAGGCA S1

D3S1358 TCTATCTGTCTATCTATCTATCTATCTATCTATCTATCTATCTATCTATCTATCTATCTATCTA S1

D3S1358 TCTATCTGTCTGTCTATCTATCTATCTATCTATCTATCTATCTATCTATCTATCTATCTA S1

FGA GGAAGGAAGGAGAAAGAAAGAAAGAAAGAAAGAAAGAAAGAAAGAAAGAAAGAAAGAAAGAGAAAAAAGAAAGAAAGAAA S1

FGA GGAAGGAAGGAGAAAGAAAGAAAGAAAGAAAGAAAGAAAGAAAGAAAGAAAGAAAGAAAGAAAGAAAGAAAGAGAAAAAAGAAAGAAAGAAA S1

D5S818 ATCTATCTATCTATCTATCTATCTATCTATCTATCTATCTATCT S1

D5S818 ATCTATCTATCTATCTATCTATCTATCTATCTATCTATCTATCTATCT S1

CSF1PO ATCTATCTATCTATCTATCTATCTATCTATCTATCTATCTATCTATCT S1

CSF1PO ATCTATCTATCTATCTATCTATCTATCTATCTATCTATCT S1

D7S820 TATCTATCTATCTATCTATCTATCTATCTATCTATCTATCTATC S1

D7S820 TATCTATCTATCTATCTATCTATCTATCTATCTATCTATCTATCTATC S1

D8S1179 TCTATCTATCTATCTATCTATCTATCTATCTATCTATCTATCTATCTATCTA S1

D8S1179 TCTATCTATCTGTCTATCTATCTATCTATCTATCTATCTATCTATCTATCTATCTATCTATCTA S1

D10S1248 GGAAGGAAGGAAGGAAGGAAGGAAGGAAGGAAGGAAGGAAGGAAGGAAGGAAGGAAGGAAGGAA S1

D10S1248 GGAAGGAAGGAAGGAAGGAAGGAAGGAAGGAAGGAAGGAAGGAAGGAAGGAAGGAA S1

TH01 AATGAATGAATGAATGAATGAATGAATGAATGAATG S1

TH01 AATGAATGAATGAATGAATGAATGATGAATGAATGAATG S1

vWA TAGATAGATAGATAGATAGATAGATAGATAGATAGATAGATAGACAGACAGACAGACAGATAGA S1

vWA TAGATAGATAGATAGATAGATAGATAGATAGATAGATAGATAGATAGATAGACAGACAGACAGACAGATAGA S1

D12S391 AGATAGATAGATAGATAGATAGATAGATAGATAGATAGATAGATAGATAGATAGACAGACAGACAGACAGACAGACAGACAGACAGAT S1

D12S391 AGATAGATAGATAGATAGATAGATAGATAGATAGATAGATAGATAGATAGATAGACAGACAGACAGACAGACAGACAGACAGACAGAC S1

D13S317 TATCTATCTATCTATCTATCTATCTATCTATCTATCTATCTATC S1

D13S317 TATCTATCTATCTATCTATCTATCTATCTATCTATCTATCTATCTATCTATC S1

D16S539 GATAGATAGATAGATAGATAGATAGATAGATAGATAGATAGATA S1

D18S51 AGAAAGAAAGAAAGAAAGAAAGAAAGAAAGAAAGAAAGAAAGAAAGAAAGAAAGAAAGAAAGAA S1

D18S51 AGAAAGAAAGAAAGAAAGAAAGAAAGAAAGAAAGAAAGAAAGAAAGAAAGAA S1

D19S433 CCTTCCTTCCTTCCTTCCTTCCTTCCTTCCTTCCTTCCTTCCTTCCTTCCTACCTTCTTTCCTT S1

D19S433 CCTTCCTTCCTTCCTTCCTTCCTTCCTTCCTTCCTTCCTTCCTTCCTTCCTTCCTTCCTTCCTACCTTTTCCTT S1

D21S11 TCTATCTATCTATCTATCTGTCTGTCTGTCTGTCTGTCTGTCTATCTATCTATATCTATCTATCTATCATCTATCTATCCATATCTATCTATCTATCTATCTATCTATCTATCTATCTATCTATCTATCTA S1

D22S1045 ATTATTATTATTATTATTATTATTATTATTATTATTATTACTATTATT S1

# Input data of haplotype for HG003 (S2)

#CODIS core STR loci for HG003(S2)

#Marker_Name Sample_haplotype SampleID

D1S1656 CCTATCTATCTATCTATCTATCTATCTATCTATCTATCTATCTATCTATCTATCTATCTATCTA S2

D1S1656 CCTATCTATCTATCTATCTATCTATCTATCTATCTATCTATCTATCTATCTA S2

TPOX AATGAATGAATGAATGAATGAATGAATGAATGAATGAATGAATG S2

TPOX AATGAATGAATGAATGAATGAATGAATGAATG S2

D2S441 TCTATCTATCTATCTATCTATCTATCTATCTATCTATCTATCTA S2

D2S1338 GGAAGGAAGGAAGGAAGGAAGGAAGGAAGGAAGGAAGGAAGAAAGGAAGGAAGGCAGGCAGGCAGGCAGGCAGGCAGGCA S2

D2S1338 GGAAGGAAGGACGGAAGGAAGGAAGGAAGGAAGGAAGGAAGGAAGGAAGGAAGGAAGGAAGGAAGGAAGGAAGGCAGGCAGGCAGGCAGGCAGGCA S2

D3S1358 TCTATCTGTCTGTCTATCTATCTATCTATCTATCTATCTATCTATCTATCTATCTATCTA S2

D3S1358 TCTATCTGTCTATCTATCTATCTATCTATCTATCTATCTATCTATCTATCTATCTATCTATCTA S2

FGA GGAAGGAAGGAGAAAGAAAGAAAGAAAGAAAGAAAGAAAGAAAGAAAGAAAGAAAGAAAGAAAGAAAGAAAGAGAAAAAAGAAAGAAAGAAA S2

FGA GGAAGGAAGGAGAAAGAAAGAAAGAAAGAAAGAAAGAAAGAAAGAAAGAAAGAAAGAAAGAAAGAAAGAAAGAAAGAGAAAAAAGAAAGAAAGAAA S2

D5S818 ATCTATCTATCTATCTATCTATCTATCTATCTATCTATCTATCT S2

D5S818 ATCTATCTATCTATCTATCTATCTATCTATCTATCTATCTATCTATCT S2

CSF1PO ATCTATCTATCTATCTATCTATCTATCTATCTATCTATCTATCT S2

CSF1PO ATCTATCTATCTATCTATCTATCTATCTATCTATCTATCTATCTATCT S2

D7S820 TATCTATCTATCTATCTATCTATCTATCTATCTATCTATC S2

D7S820 TATCTATCTATCTATCTATCTATCTATCTATCTATCTATCTATC S2

D8S1179 TCTATCTGTCTATCTATCTATCTATCTATCTATCTATCTATCTATCTATCTA S2

D8S1179 TCTATCTATCTGTCTATCTATCTATCTATCTATCTATCTATCTATCTATCTATCTATCTATCTA S2

D10S1248 GGAAGGAAGGAAGGAAGGAAGGAAGGAAGGAAGGAAGGAAGGAAGGAAGGAAGGAA S2

TH01 AATGAATGAATGAATGAATGAATGATGAATGAATGAATG S2

vWA TAGATAGATAGATAGATAGATAGATAGATAGATAGATAGATAGATAGATAGACAGACAGACAGACAGATAGA S2

vWA TAGATAGATAGATAGATAGATAGATAGATAGATAGATAGATAGATAGACAGACAGACAGACAGATAGA S2

D12S391 AGATAGATAGATAGATAGATAGATAGATAGATAGATAGATAGATAGATAGATAGACAGACAGACAGACAGACAGACAGACAGACAGAC S2

D12S391 AGATAGATAGATAGATAGATAGATAGATAGATAGACAGACAGACAGACAGACAGACAGAT S2

D13S317 TATCTATCTATCTATCTATCTATCTATCTATCTATCTATCTATCTATC S2

D13S317 TATCTATCTATCTATCTATCTATCTATCTATCTATCTATCTATCTATCTATC S2

D16S539 GATAGATAGATAGATAGATAGATAGATAGATAGATAGATAGATA S2

D16S539 GATAGATAGATAGATAGATAGATAGATAGATAGATA S2

D18S51 AGAAAGAAAGAAAGAAAGAAAGAAAGAAAGAAAGAAAGAAAGAAAGAAAGAAAGAA S2

D18S51 AGAAAGAAAGAAAGAAAGAAAGAAAGAAAGAAAGAAAGAAAGAAAGAAAGAA S2

D19S433 CCTTCCTTCCTTCCTTCCTTCCTTCCTTCCTTCCTTCCTTCCTTCCTTCCTACCTTCTTTCCTT S2

D21S11 TCTATCTATCTATCTATCTGTCTGTCTGTCTGTCTGTCTGTCTATCTATCTATATCTATCTATCTATCATCTATCTATCCATATCTATCTATCTATCTATCTATCTATCTATCTATCTATCTATCTATCTA S2

D21S11 TCTATCTATCTATCTATCTGTCTGTCTGTCTGTCTGTCTGTCTATCTATCTATATCTATCTATCTATCATCTATCTATCCATATCTATCTATCTATCTATCTATCTATCTATCTATCTATCTA S2

D22S1045 ATTATTATTATTATTATTATTATTATTATTATTATTATTACTATTATT S2

D22S1045 ATTATTATTATTATTATTATTATTATTATTATTATTACTATTATT S2
